# Cross-sectional study on community pharmacists’ behaviours in providing travel health services in Türkiye: a structural equation modelling approach

**DOI:** 10.1136/bmjopen-2025-112047

**Published:** 2026-05-15

**Authors:** Miray Arslan, Ayşe Çiğdem Şehitoğlu, Gülbin Özçelikay

**Affiliations:** 1 Department of Pharmacy Management, Faculty of Pharmacy, Van Yüzüncü Yıl Üniversitesi, Van, Turkey; 2 Department of Pharmacy Management, Faculty of Pharmacy, Ankara Universitesi, Ankara, Turkey

**Keywords:** Pharmacology, Public health, Pharmacists

## Abstract

**Abstract:**

**Objectives:**

This study aimed to evaluate the behaviours of community pharmacists in Türkiye regarding the provision of travel health services (THS) and to identify the determinants of these behaviours using the theory of planned behaviour (TPB).

**Design:**

A cross-sectional descriptive study.

**Setting:**

Online nationwide survey conducted in Türkiye.

**Participants:**

The study included 145 Turkish community pharmacists with at least 5 years of professional experience who had provided THS to at least one patient in the previous year.

**Interventions:**

A theory-based measurement tool was developed and validated according to the TPB framework. Data were collected via self-administered online questionnaires between May 2024 and July 2024. Structural equation modelling was employed to analyse the factors influencing pharmacists’ behaviours and intentions towards THS.

**Results:**

The structural equation model demonstrated an acceptable fit with the empirical data. Pharmacists’ intentions to provide THS were significantly influenced by subjective norms (β=0.19, p<0.05) and perceived behavioural control (PBC) (β=0.49, p<0.001). PBC was identified as the strongest predictor of intention. Furthermore, pharmacists’ intentions significantly and positively influenced their actual behaviour in providing THS (β=0.49, p<0.001).

**Conclusions:**

The findings confirm that providing THS is a suitable and essential role for community pharmacists in Türkiye. Since PBC is the primary driver of intention, policy interventions should focus on empowering pharmacists through specialised training and expanding their professional autonomy to better integrate travel health into community pharmacy practice.

**STRENGTHS AND LIMITATIONS OF THIS STUDY**

- Community pharmacists’ travel health behaviours were evaluated using structural equation modelling within the theory of planned behaviour framework.
- A specialised, theoretically robust measurement tool was developed and validated for this field.
- The cross-sectional design limits the ability to establish a causal relationship.

## Introduction

 Travel is a significant aspect of human life both in Türkiye and worldwide. Consequently, health risks related to travel are increasing, and the concept of travel health is coming to the forefront.^1^,^6^ Travel health services (THS) are an interdisciplinary field that has a crucial role in ensuring travellers’ health and safety before, during and after their trips.^5^,^12^ Additionally, providing THS (PTHS) benefits not only travellers’ health but also the health of residents of the countries they visit, especially by preventing the spread of infectious diseases.^13^

Studies show that pharmacists can provide comprehensive THS.^14^,^16^ Community pharmacies are well-positioned to provide THS, particularly for travel vaccines, COVID-19 vaccines, medications and health advice, due to their convenient locations, established patient portfolios and collaboration with physicians.^117^,^21^ Pharmacists help protect the health of travellers and avoid life-threatening risks by providing primarily medication counselling, interpreting patient symptoms, referring travellers in need of prescription drug treatments to their family physicians or a travel clinic, supplying medications, ensuring patients have safe and adequate access to their medications, and increasing access to preventive measures.^22^ Community pharmacies are an essential point of contact for travellers, not only for providing prescription medications but also for advice on non-prescription products that may be needed while travelling, such as antidiarrheal and sunscreen products, antiemetics, insect repellents and medical supplies.^15 23 24^ Additionally, pharmacists providing pretravel medical advice to travellers with known pre-existing medical conditions and monitoring for travellers with special needs (eg, pregnant women, diabetic patients) help protect patients’ health during travel by preventing their existing chronic diseases from flaring up.^25 26^

In Türkiye, THS are primarily administered by the General Directorate of Border and Coastal Health, which is affiliated with the Ministry of Health. These services aim to protect and inform citizens travelling abroad about the health risks in the regions they will visit. Within this scope, services such as travel vaccinations, drug-based protection and individual counselling are offered. However, there is no regulation governing pharmacists’ provision of these services, and, to the best of the authors’ knowledge, no data are available on the extent to which pharmacists provide THS. Community pharmacies are recognised for their high level of public trust and stand as the most accessible healthcare providers for travellers.^1 27 28^ While the importance of integrating THS into general pharmacy practices is well documented in the literature, there remains a gap in the detailed evaluation of pharmacists’ behaviours in travel health. To improve Turkish community pharmacists’ role in PTHS, it is not enough to know current practices; understanding the root causes of these behaviours is necessary. Therefore, this study aims to analyse the elements of attitude, subjective norms (SN) and perceived behavioural control that shape pharmacists’ intentions to provide THS within the theory of planned behaviour (TPB) framework. Several studies have evaluated and modelled pharmacists’ behaviour in different areas using the TPB. According to the literature, the TPB is among the most widely used theories in pharmacy practice research.^29^,^31^

In this regard, the main objectives of this study are: (1) to identify the attitude, SN and perceived behavioural control (PBC) of the Turkish community pharmacists’ behavioural intention to provide THS; (2) to determine the predictors for the Turkish community pharmacists’ behavioural intention to provide THS; (3) to model community pharmacists’ behaviour in PTHS.

## Materials and methods

### Theoretical framework and hypothesis

The theoretical background of the current study is based on the theory of planned behaviour, developed by Icek Ajzen in 1985. In the TPB, individuals’ attitudes, SNs and perceived behavioural control are used to explain behavioural intentions, and intentions are the fundamental antecedents of actual behaviour.^32 33^ Attitudes towards behavioural intention refer to an individual’s positive or negative evaluation of it.^33^ Community pharmacists’ attitudes towards providing THS were measured using eight items (ie, *Travel health services provided by pharmacists mitigate potential health risks, PTHS by pharmacists strengthens pharmacists’ image, PTHS by pharmacists strengthens the pharmacy economy*). Hence, the first hypothesis of the study was established as: (H_1_) community pharmacists’ attitudes towards PTHS positively affect their intentions towards PTHS.

Ajzen defined SN as the subject’s perception of social pressure from individuals who are significant to them in performing the behaviour in question.^33^ In Turkey, there is no material or financial support for pharmacists to provide THS; however, Turkish regulations define pharmacists’ roles comprehensively, and, according to these, pharmacists are known to play a role in travel health. In this study, perceived social support is evaluated within the framework of SNs using 4 items focused on community, government, colleagues and health professionals’ support (eg, ‘The government supports me in PTHS’). In this regard, the second hypothesis of the study is (H_2_) Community pharmacists’ SNs towards PTHS positively affect their intentions toward PTHS.

PBC measures the individual’s confidence in their ability to perform the behaviour. It predicts the probability of a successful behavioural attempt by accounting for non-volitional factors that are barriers to the individual.^34^ Respondents indicated, through eight items, how confident they were in their ability to provide THS (eg, *I have employees/employees who will help me in PTHS; It is easy for me to provide PTHS*). So, the third hypothesis was as follows: (H_3_): community pharmacists’ perceived behavioural control related to PTHS positively affects their intentions toward PTHS.

In the study, community pharmacists’ intention to provide THS was measured using four items (ie, *I intend/plan/am willing/am thinking to provide THS*). Intention, the strongest antecedent of behaviour, triggers action when suitable opportunities arise.^35^ To test this relationship in the theoretical framework, the last hypothesis was formulated by including the actual behavioural dimension in the proposed model: (H_4_) Community pharmacists’ intentions towards PTHS positively affect their behaviour in PTHS.

**Figure 1:**
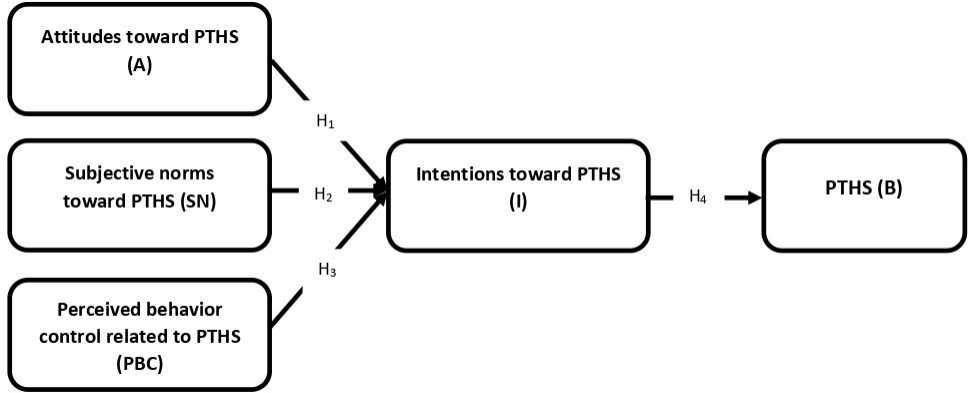
Research model for community pharmacists’ behaviours in PTHS (PTHS: providing travel health services).

To test the mentioned hypotheses, a model of *community pharmacists’ behaviour in PTHS* has been proposed (figure 1). The research model defines how latent variables (attitude, SN, perceived behavioural control and intention) are measured through observed (behaviour) variables. The structural model defines the causal relationships and associations between latent and observed variables.

### Measurement tool

A measurement tool was developed to explore Turkish community pharmacists’ behaviour in providing THS. Based on the basic TPB constructs and related literature,^3336^,^39^ an item pool including 38 items was prepared. Items were evaluated by Lawshe’s method.^38^ The content validity ratio (CVR) value was calculated for each item, and items with negative or zero CVR values were eliminated from the pool. At the α=0.05 significance level, for five experts, the minimum CVR should be 0.99.^40^ Hence, two items with a CVR value below 0.99 were removed from the measurement tool. The measurement tool consisted of 36 Turkish items, evaluated using a 5-point Likert scale (please see the online supplemental information for the Turkish version of the measurement tool).

Finally, demographic questions were added to the questionnaire to investigate gender, educational level, work experience, the number of patients who need THS per year, the services commonly provided under travel health and participants’ status regarding a travel health course/training/education programme. Twelve PhD students in the Pharmacy Management programme, who were working as community pharmacists and were not among the authors, piloted the questionnaire online. Following feedback from the pilot study, some of the items were revised. Participants in the pilot study reported a completion time of 6–8 min, and based on this, it was predicted that the completion time would be approximately 10 min. However, to avoid any loss of information, no time limit was applied during the questionnaire administration.

### Study population and data collection

The study population consisted of Turkish community pharmacists with at least 5 years of community pharmacy experience who had provided THS to at least one patient in the previous year. The survey was conducted between May and June 2024 via Google Forms. The questionnaire link was shared with the Turkish Pharmacist Association and the Pharmacists Chambers in Türkiye, and, with their support, it reached community pharmacists. There are 52 Pharmacist Chambers affiliated with the Turkish Pharmacists Association, and these chambers distributed the questionnaires to pharmacists via email. The number of pharmacists to be invited from each chamber was planned in proportion to the number of members in each chamber, and chamber managers emailed the questionnaire link to randomly selected community pharmacists. Chamber managers also sent a reminder email.

The questionnaire was voluntary and offered no incentives to participants. Patients and the public were not involved in any part of this study. This is a web-based questionnaire comprising 10 pages. The first page provides brief information about the study. Pharmacists were invited to give consent on the second page. The following pages include 4 to 10 questions per page. The survey items were randomised to avoid biases. Respondents could review and change their answers. A Completely Automated Public Turing test to tell Computers and Humans Apart (CAPTCHA) was included at the beginning of the questionnaire to determine the legitimacy of responses.

#### Sample size calculation

According to official records, approximately 20 000 community pharmacists with 5 years of experience were estimated to be practising in Turkey during the study period; however, no records are available on the number of pharmacists providing THS. Therefore, the minimum sample size was calculated by “ $n=\left(Z_{1-\infty /2}^{2}*p*(1-p)\right)/e^{2}$” with a 90% confidence level and 0.05 sampling error (n: sample size; Z: Z-value (corresponding to confidence level); p: estimated population proportion; e: margin of error). Accordingly, the minimum sample size was determined to be 96. To improve the reliability and validity of the study, questionnaires were emailed to pharmacists, ensuring a sample size at least 10 times the minimum required.

### Data analysis

As stated in previous sections, developing a measurement tool is one of the study’s aims. Therefore, according to the relevant literature, the combined use of exploratory factor analysis (EFA) and confirmatory factor analysis (CFA) was chosen to get more consistent results.^41 42^ So, following the demographic statistics, the EFA was conducted using the Varimax rotation method. Then, the CFA was performed to check the factorial validity of the factors. The reliability of the measurement tool was determined by calculating Cronbach’s alpha and composite reliability (CR) coefficients. Additionally, convergent and discriminant validity of the measurement tool were assessed using factor loadings, the correlation matrix among factors, average variance extracted (AVE) and the square root of AVE (√AVE).^42^,^45^ Finally, the structural equation model (SEM) was used to test hypotheses. Goodness-of-fit indices were used to assess the fit of the CFA and SEM IBM Statistical Package for Social Sciences V.22 and LISREL V.8.80 were used to conduct the analysis.

The results of the study were reported in accordance with the Checklist for Reporting Results of Internet Surveys (CHERRIES).^46^

## Results

### Sociodemographic results

960 community pharmacists were invited to participate in the study; 156 did. 11 questionnaires were excluded from the analysis due to missing responses, as participants did not complete many questions. The analysis continued with the responses from 145 participants. Therefore, the completion rate was nearly 93%, and the response rate was 15%.

Ninety-four respondents were female, 50 were male and 1 preferred not to state gender. Nearly 81% of the community pharmacists had bachelor’s degrees and 15 had master’s degrees. Only nine respondents had participated in a course/training/education programme about travel health. About 40% of the respondents stated that they provide THS for 10 or fewer patients per year, 35% of them provide THS for 10 to 20 patients, 10% of them provide THS for 20 to 30 patients and 15% of them provide THS for more than 30 patients.

Furthermore, it has been revealed that pharmacists provide consultancy services, especially on diarrhoea/constipation, sunburns and motion sickness, within the scope of THS (table 1).

**Table 1:** Consultancy services provided by pharmacists within the scope of THS (n=156)

Disease	n (%)
Diarrhoea/constipation	143 (91.6)
Sunburns	137 (87.8)
Motion sickness	134 (85.9)
Cold	118 (75.6)
Insect bites and stings	118 (75.6)
Heatstroke	110 (70.5)
Fever	109 (69.8)
Food poisoning	95 (60.9)
Infectious/contagious diseases	93 (59.6)
Chronic diseases	90 (57.7)
Paediatric diseases	78 (50.0)
Pregnancy and lactation	74 (47.4)
Vaccines	73 (46.8)
Sexually transmitted diseases	60 (38.5)
Oral health	57 (36.6)
Other	10 (6.4)

THS, travel health services.

### Results of the EFA

The Kaiser-Meyer-Olkin value was 0.895, indicating that the sample size was adequate for conducting the EFA. Factors were extracted using principal component analysis with a varimax rotation. Six items (with loadings below 0.5) were removed from the EFA based on factor loadings and eigenvalues. A five-factor structure was obtained with 30 items. The factors were labelled as (1) attitudes towards PTHS (A), (2) SNs towards PTHS (SN), (3) perceived behavioural control related to PTHS (PBC), (4) intentions towards PTHS (I) and (v) behaviour in providing THS (B). The five-factor structure explained 78.253% of the total variance. The factor structures and factor loadings are listed in table 2.

**Table 2:** Results of the EFA (PTHS: providing travel health services)

Items	Median	Factor loadings				
		B	PBC	A	I	SN
b5. I advise my patients who are going to travel about the medications they may need during their travel.	4.00	0.863				
b6. I advise my patients who are going to travel about the medications they use.	4.00	0.858				
b7. I advise my patients who are going to travel about the non-pharmaceutical products they use.	4.00	0.838				
b8. I advise my patients who are going to travel about the non-pharmaceutical products they may need during their travel.	4.00	0.830				
b2. I advise my patients who are going to travel about the health problems they may encounter during their travel.	4.00	0.795
b3. I do immunisation counselling for my patients who are going to travel.	4.00	0.786				
b1. I do a health risk assessment for my patients who are going to travel.	4.00	0.770
b9. I advise my patients who are going to travel about the protective materials they may need during their travel.	4.00	0.762
b4. I advise my patients who are going to travel about the first aid information they may need during their travel.	4.00	0.715
pbc7. I have the skills for PTHS.	4.00		0.849			
pbc6. I have the knowledge of PTHS.	4.00		0.848			
pbc3. I have the education for PTHS.	4.00		0.834			
pbc9. I can cooperate with other health professionals while PTHS.	4.00	0.756
pbc2. I have the confidence for PTHS.	4.00		0.749			
pbc8. I can access up-to-date information about travel health.	4.00	0.725
pbc4. I have employees/employees who will help me in PTHS	4.00	0.716
pbc1. It is easy for me for PTHS.	4.00	0.662
a8. PTHS by pharmacists strengthens community health.	5.00			0.912		
a7. Travel health services provided by pharmacists mitigate potential health risks.	5.00			0.908		
a5. PTHS by pharmacists strengthens the pharmacists’ image.	5.00			0.907		
a4. It is important that pharmacists advise travellers about things that should be put in a first aid kit.	5.00	0.836
a3. It is valuable for pharmacists to advise travellers about immunisation before visiting risky destinations.	5.00	0.815
a6. PTHS by pharmacists strengthens the pharmacy economy.	5.00			0.790		
i2. I plan to PTHS.	4.00				0.871	
i3. I am willing to PTHS.	4.00				0.848	
i1. I intend to PTHS.	4.00				0.823	
i4. I am thinking to PTHS.	4.00				0.787	
sn2. The government supports me in PTHS.	3.00					0.883
sn4. Other health professionals support me in PTHS.	4.00					0.810
sn1. The community supports me in PTHS.	4.00					0.511
Cumulative variance explained (%)		22.491	41.846	59.336	71.117	78.253

EFA, exploratory factor analysis; PBC, perceived behavioural control; SN, subjective norms.

Thus, the theoretical outline of the model was developed by identifying the latent structures to which the dataset’s variables are clustered using EFA. In the next stage, the CFA was used to test how well this outline structure fits the data.

### Results of the CFA

The CFA results indicate that all variables were significant (p<0.05). The factor loadings for all items ranged from 0.67 to 0.93 (online supplemental figure 1). The 30-item solution showed an acceptable fit to data from the validation sample, thus confirming its structure (table 3).^44 47 48^

**Table 3:** Goodness-of-fit indices’ acceptable limits and goodness-of-fit indices for the developed CFA model^44 47 48^

Fit measures	Developed model	Acceptable fit
χ^2^/d.f.	2.304	χ^2^/d.f. ≤ 3
Root mean square error of approximation (RMSEA)	0.095	RMSEA≤0.10
Root mean square residual (RMR)	0.064	RMR≤0.10
Standardised root mean square residual (SRMR)	0.069	SRMR≤0.10
Incremental fit index (IFI)	0.96	IFI>0.90
Comparative fit index (CFI)	0.96	CFI>0.90
Normed fit index (NFI)	0.93	NFI>0.95
Non-normed fit index (NNFI)	0.95	NNFI>0.95

CFA, confirmatory factor analysis.

Before examining the relationships among variables, the reliability and validity of the measurement tool developed using CFA were first established to ensure error-free measurement.

### Validity and reliability

The reliability of the measurement tool was verified using Cronbach’s alpha, which indicates the internal consistency. As shown in table 4, these values exhibit high reliability for these factors.^44 45^

**Table 4:** Correlation matrix-related factors, Cronbach’s alpha (α), average variance extracted (AVE) and composite reliability (CR) coefficients

	A	SN	I	PBC	B
A	1				
SN	0.44	1			
I	0.38	0.47	1		
PBC	0.47	0.52	0.62	1	
B	0.39	0.31	0.48	0.58	1
Cronbach alpha (α)	*0.940*	*0.808*	*0.947*	*0.949*	*0.949*
AVE	*0.788*	*0.604*	*0.819*	*0.713*	*0.684*
The square root of the AVE	*0.888*	*0.777*	*0.905*	*0.844*	*0.827*
CR coefficient	*0.957*	*0.819*	*0.948*	*0.952*	*0.951*

PBC, perceived behavioural control; SN, subjective norms.

For validity, factor loadings were first examined, and it was found that the standardised factor loadings for nearly all items were above 0.670. Then, the AVE values and CR coefficients were calculated and are presented in table 4.

According to table 4, the AVE values for all factors are greater than 0.5 and the CR coefficients are higher than 0.6, which are accepted as the cut-off points.^43 45^ The square roots of the AVEs were calculated to evaluate discriminant validity and were found to exceed the correlation estimates between the related factors (0.5). These findings indicate that the developed measurement tool has appropriate convergent and discriminant validities.^43 45^

### Results of the SEM

**Figure 2:**
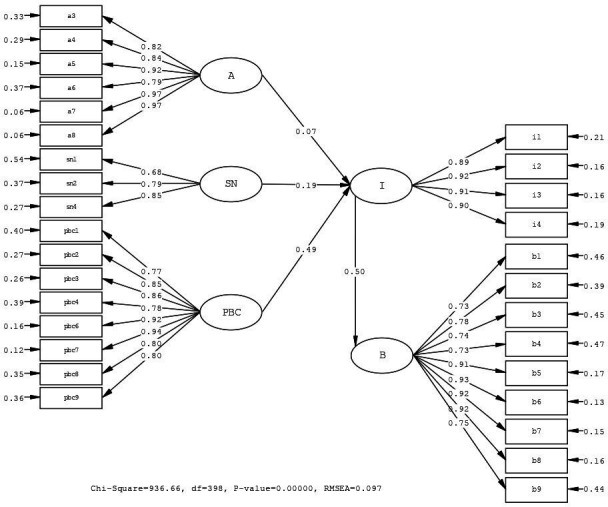
LISREL V.8.80 output for community pharmacists’ behaviour in the PTHS model. PBC, perceived behavioural control; PTHS, providing travel health services; RMSEA, root mean square error of approximation; SN, subjective norms.

The measurement model validated in the CFA phase serves as the structural framework for the SEM model. This allowed testing of cause-and-effect relationships between variables, free of measurement errors. The SEM is performed using the Maximum Likelihood method to investigate relationships among latent variables and test the research hypothesis. The path model obtained from the SEM is given in figure 2. The proposed model performs an acceptable fit (χ^2^/df: 2.353; root mean square error of approximation: 0.097; root mean square residual: 0.10; standardised root mean square residual: 0.10; incremental fit index: 0.95; comparative fit index: 0.95; non-normed fit index: 0.95) for the data.

Standardised parameter estimates for paths, t-values, structural equations and reduced-form equations related to the community pharmacists’ PTHS model are presented in table 5.

**Table 5:** Results of Structural Equation Model (SEM)

Hypotheses	Paths	Standardised parameter estimation values	t-values	Results
H_1_	(A)→(I)	0.07	0.92	Rejected
H_2_	(SN)→(I)	0.19	2.03	Confirmed
H_3_	(PBC)→(I)	0.49	5.32	Confirmed
H_4_	(I)→(B)	0.50	5.68	Confirmed
Structural equations	I=0.07*A+0.19*SN+0.49*PBC B=0.50*I		R^2^=0.43 R^2^=0.25	
Reduced equations	I=0.07*A+0.19*SN+0.49*PBC B=0.037*A+0.094*SN+0.25*PBC		R^2^=0.43 R^2^=0.11	

PBC, perceived behavioural control; SN, subjective norms.

There was no significant relationship between attitudes and intention towards PTHS. Hence, the first research hypothesis was rejected, and others were confirmed. The coefficients of determination for the structural equations were 0.43 and 0.25, respectively. These values show that independent latent variables ‘A’, ‘SN’ and ‘PBC’ explained 43% of the variance in the dependent latent variable ‘I’. Additionally, ‘I’ explained 25% of the variance in the dependent latent variable ‘B’.

## Discussion

This study was conducted among Turkish community pharmacists with 5 years of community pharmacy experience and who provided THS to at least one patient last year. The study’s results make two primary contributions to the literature. The first is developing a valid and reliable measurement tool to investigate pharmacists’ behaviours and their antecedents regarding THS, and the second is presenting a PTHS model. To the authors’ knowledge, this is the first study to comprehensively evaluate community pharmacists’ behaviours regarding PTHS. Furthermore, this study represents the first nationwide analysis of pharmacists’ travel health practices in Türkiye. The results may shed light on the roles of community pharmacists and policymakers in THS, increasing awareness of this issue and the quality of THS provided by community pharmacists.

It was observed that the number of patients to whom pharmacists provided annual THS was similar to the studies in the literature.^37 49 50^ In parallel, Hurley-Kim *et al* reported that many pharmacists in the USA provide THS.^28^ Additionally, diarrhoea/constipation, sunburn and motion sickness are reported as the most common topics for THS in the related literature.^1937 49^,^51^ In addition, the literature addresses pharmacists’ knowledge and skills in administering vaccines within the scope of THS. The role of pharmacists in travel health is generally limited to vaccination today, and pharmacists must be trained on this subject to prevent this misconception.^52^ Although pharmacists in Türkiye believe that offering vaccination services benefits both their pharmacies and their patients, they do not have the authority to administer vaccines.^1 37 51^ Similarly, when the median values for the items measuring attitudes towards the PTHS factor are examined, it is evident that community pharmacists have positive attitudes towards PTHS. However, in the model presented in this study, no significant relationship between attitude and intention was observed, contrary to TPB predictions. This finding was quite surprising. As Bagozzi^53^ stated, in some cases, a positive attitude alone may not be sufficient for motivation, and a lack of motivation or desire may affect the relationship between attitude and intention. Hence, it is thought that more initiatives are needed to increase community pharmacies’ motivation regarding THS in Türkiye.

The systematic review conducted by Abdul Kadir *et al* revealed that doctors do not adequately support pharmacists in PTHS, and this situation is perceived as a barrier to pharmacists providing these services.^54^ From a different perspective, Heslop *et al* argue that travellers are supporting pharmacists in PTHS. In this study, it is seen that other health professionals and society, apart from the government, support pharmacists in this regard.^37^ In addition, the presented model determined that SNs on this issue positively affect intention.

Laliberté *et al* revealed that although pharmacists are aware of their essential roles in providing health services, they lack confidence in their ability to deliver these services due to certain barriers.^55^ Heslop *et al*, Thidrickson and Goodyer and El-Kurdi *et al* stated that pharmacists found some issues (time, staff, cost, etc) as barriers to PTHS.^37 56 57^ Kc and Juneja reported that most pharmacists are interested in PTHS, given their special education needs in this area.^1 16^ On the contrary, the current study’s findings reveal that pharmacists’ perceived behavioural control towards this service is high, that they are confident in providing it in this context, and that they do not see the barriers presented in the literature as barriers to them. As Özkaya and Özçelikay stated, community pharmacists with higher self-efficacy perceive barriers as opportunities for success.^58^ In this context, the finding that pharmacists’ perceived behavioural control has a positive effect on the intention to provide THS in the presented model is expected.

In many studies examining the provision of pharmaceutical care services, it has been found that pharmacists’ intentions positively affect their behaviour.^30 59 60^ In line with the literature, this study also shows that the intention towards PTHS positively affects pharmacists’ provision of this service. Within the behavioural factor, it is observed that pharmacists frequently advise their travelling patients on a range of issues, from non-pharmaceutical products to immunisations.

The limitations of this study included its quantitative design and the use of a self-administered online questionnaire. Future research incorporating qualitative methodologies is recommended to explore why pharmacists’ attitudes did not significantly impact their behaviour. The use of self-administered online questionnaires may introduce social desirability bias, potentially affecting the objectivity of reported behaviours. Also, there are limitations on the response rate and sample size. Although aiming to reach a highly specific professional group—community pharmacists with at least 5 years of experience who are actively involved in travel health—led to a low response rate (15%). The final sample size (n=145) met the statistical requirements for SEM, ensuring the robustness of the analysis.

### Conclusions

A measurement tool was developed and validated within the theory of planned behaviour (TPB) framework to evaluate the behaviours of community pharmacists in PTHS. The applied stepwise analysis approach—progressing through Exploratory Factor Analysis, Confirmatory Factor Analysis, and Structural Equation Model—ensures that the results obtained are not merely superficial correlations but the product of a theoretically robust structure free of measurement error. This rigorous validation process confirms that the non-significant relationship found between attitudes and intention is an empirically sound finding rather than a measurement artefact. Consequently, the model’s ability to explain 43% of the variance in intention (I) and 25% of the variance in behaviour (B) provides a reliable foundation for practical interventions. The provided *Community Pharmacists’ PTHS Model* clearly showed that community pharmacists’ perceived behavioural control towards the PTHS and was the strongest predictor of their intentions towards PTHS.

The findings from this study confirm that THS is a suitable role for Turkish pharmacists. According to related literature on THS provided by community pharmacists, the vaccine-centric approach, in particular, attracts attention. However, pharmacists cannot provide vaccination services under Turkish pharmacy legislation. Also, there are no formal licensing or registration requirements, nor any incentive or training programmes for community pharmacists regarding the PTHS in Türkiye. Hence, this study also underscores the need to highlight the changing roles of pharmacists worldwide and to enact legislative changes to adapt to them. By offering opportunities and training to motivate Turkish pharmacists to provide these services, it may be possible to align their practices with international standards and achieve better outcomes for travellers.

## Supplementary material

10.1136/bmjopen-2025-112047 online supplemental file 1

10.1136/bmjopen-2025-112047 online supplemental file 2

## Data Availability

Data are available upon reasonable request.
