# Supplementary figures and images for: Cross-sectional study on community pharmacists’ behaviours in providing travel health services in Türkiye: a structural equation modelling approach
Source: BMJ Open. 2026 May 15;16(5):e112047. doi: 10.1136/bmjopen-2025-112047 (PMC13182400; doi:10.1136/bmjopen-2025-112047)

# CFA Path Model

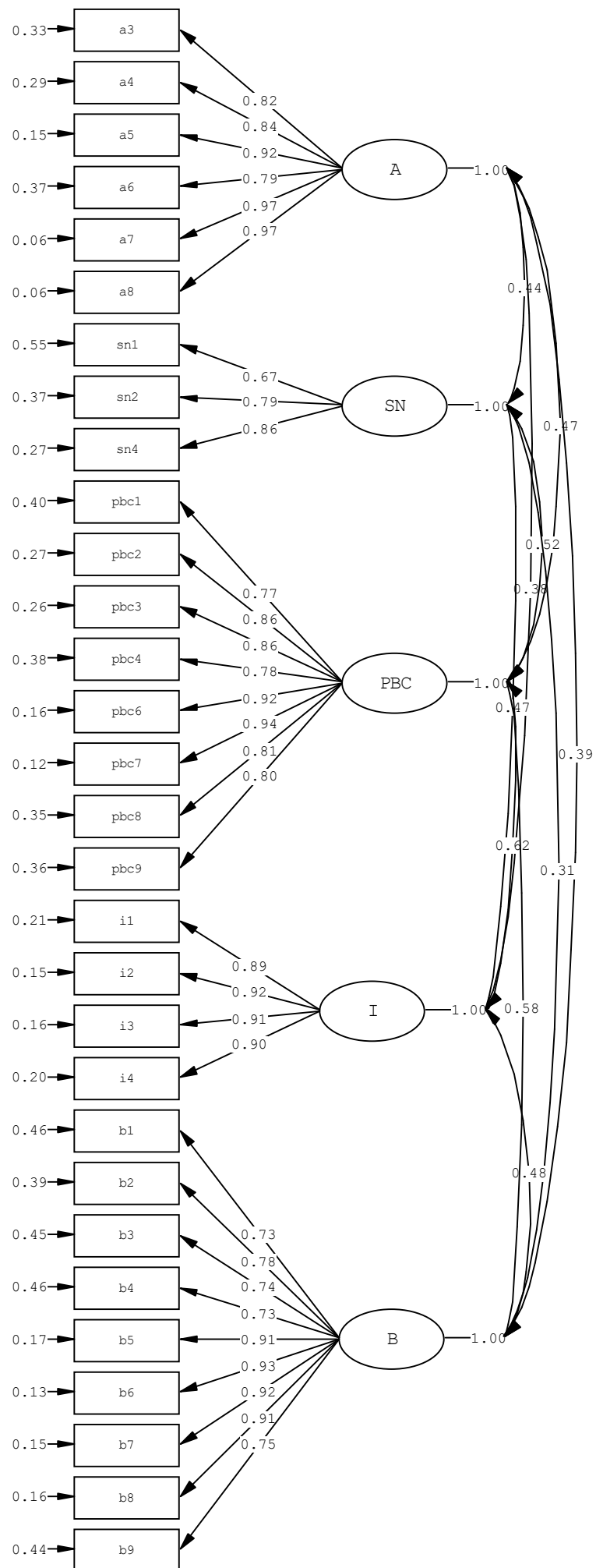

Chi-Square=910.45, df=395, P-value=0.00000, RMSEA=0.095

Supplement: online supplemental file 1 [file bmjopen-16-5-s001.pdf]
