# Supplementary material for: Cross-sectional study on community pharmacists’ behaviours in providing travel health services in Türkiye: a structural equation modelling approach
Source: BMJ Open. 2026 May 15;16(5):e112047. doi: 10.1136/bmjopen-2025-112047 (PMC13182400; doi:10.1136/bmjopen-2025-112047)
Supplement: online supplemental file 2 [file bmjopen-16-5-s002.pdf]

Toplum Eczacılarının Seyahat Sağlığı Hizmeti Sunma Davranışları

Değerli Eczacımız,

Bu araştırma, Ankara Üniversitesi ve Van Yüzüncü Yıl Üniversitesi Eczacılık Fakültesi Eczacılık İşletmeciliği Anabilim Dallarınca yürütülmektedir. Araştırma tüm Türkiye'de faaliyette olan, geçtiğimiz yıl en az bir hastaya seyahat sağlığı hizmeti sunmuş ve en az 5 yıllık tecrübeye sahip toplum eczacılarına yapılacaktır. Araştırmanın amacı, seyahat öncesinde, sırasında ve sonrasında toplum eczacılarının seyahat sağlığı hizmetlerine katkılarını belirlemektir. Bu araştırma çalışmasındaki sorulara vereceğiniz cevaplarınız, konuyla ilgili sorunların belirlenmesine ve çözüm üretilmesine katkı sağlayacaktır. Çalışmaya katılmak gönüllülük esasına dayanmaktadır; herhangi bir cezaya veya yaptırıma maruz kalmadan istediğiniz zaman araştırmayı reddedebilir, gerekçeli ya da gerekçesiz araştırmadan ayrılabilirsiniz. Toplanan veriler yalnız araştırma amacıyla kullanılacak olup, bilimsel amaçlar dışında hiçbir kurum ya da kişiye açık tutulmayacak ya da paylaşılmayacaktır. Ankette isim bildirme zorunluluğu yoktur.

Zaman ayırıp
araştırmaya katkı verdiğiniz için teşekkür ederiz.

* Zorunlu soruyu belirtir

Prof. Dr. Miray ARSLAN

Ayşe Çiğdem ŞEHİTOĞLU (Ecz. Fak. Öğrencisi)

Prof. Dr. Gülbin ÖZÇELİKAY

Çalışma ile ilgili ayrıntılı bilgi ve sorularınız için:

Ayşe Çiğdem ŞEHİTOĞLU

ecz.aysecigdemsehitoglu@gmail.com

Adsız Bölüm

1. CAPTCHA *

Uygun olanların tümünü işaretleyin.

- [ ] Ben robot değilim

2. Çalışmaya katılmayı kabul ediyor musunuz? *

Yalnızca bir şıkkı işaretleyin.

- Evet
- Hayır

Lütfen bu bölümde yer alan ifadelere katılım derecenizi 1 (Kesinlikle Katılmıyorum) - 5 (Kesinlikle Katılıyorum) aralığında derecelendiriniz.

*Anketi mobil cihazınızdan yanıtlıyorsanız soruları daha rahat görebilmek için lütfen ekranı yatay konumda tutunuz.

3. *

Her satırda yalnızca bir şıkkı işaretleyin.

	1 (Kesinlikle Katılmıyorum)	2	3	4	5 (Kesinlikl Katılıyorum
**Seyahat sağlığı hizmetlerinin sunumu eczacılar için uygun bir roldür.**	☐	☐	☐	☐	☐
**Seyahat sağlığı hizmetleri sunmamı diğer sağlık çalışanları destekler.**	☐	☐	☐	☐	☐
**Eczacıların, riskli destinasyonları ziyaret etmeden önce seyahat edenlere bağışıklama konusunda tavsiyede bulunması değerlidir.**	☐	☐	☐	☐	☐
**Eczacıların seyahat sağlığı hizmetlerini sunması olası sağlık risklerini azaltır.**	☐	☐	☐	☐	☐
**Seyahat sağlığı hizmetleri sunmamı toplum destekler.**	☐	☐	☐	☐	☐

Lütfen bu bölümde yer alan ifadelere katılım derecenizi 1 (Kesinlikle Katılmıyorum) - 5 (Kesinlikle Katılıyorum) aralığında derecelendiriniz.

*Anketi mobil cihazınızdan yanıtlıyorsanız soruları daha rahat görebilmek için lütfen ekranı yatay konumda tutunuz.

4. *

Her satırda yalnızca bir şıkkı işaretleyin.

	1 (Kesinlikle Katılmıyorum)	2	3	4	5 (Kesinlikl Katılıyorum
**Eczacıların, seyahat edecek kişiye ilk yardım çantasına koyması gereken malzemeler konusunda tavsiyelerde bulunması önemlidir.**					
**Seyahat sağlığı hizmetlerinin sunumu eczacıların imajını güçlendirir.**					
**Eczacıların seyahat sağlığı hizmetlerini sunması olası sağlık risklerini azaltır.**					
**Seyahat sağlığı hizmetleri sunmamı meslek örgütüm destekler.**					
**Eczacıların seyahat sağlığı hizmetlerini sunması toplum sağlığını güçlendirir.**					
**Seyahat sağlığı hizmetleri sunmamı devlet destekler.**					
**Seyahat sağlığı hizmetlerinin sunumu eczane ekonomisini güçlendirir.**					

Lütfen bu bölümde yer alan ifadelere katılım derecenizi 1 (Kesinlikle Katılmıyorum) - 5 (Kesinlikle Katılıyorum) aralığında derecelendiriniz.

*Anketi mobil cihazınızdan yanıtlıyorsanız soruları daha rahat görebilmek için lütfen ekranı yatay konumda tutunuz.

5. *

Her satırda yalnızca bir şıkkı işaretleyin.

	1 (Kesinlikle Katılmıyorum)	2	3	4	5 (Kesinlikl Katılıyorum
**Seyahat sağlığı hizmetlerini sunabilecek güvene sahibim.**					
**Seyahat sağlığı hizmetlerini sunabilecek bilgi birikimine sahibim.**					
**Seyahat sağlığı hizmetlerini sunmaya istekliyim.**					
**Seyahat sağlığı hizmetlerini sunmaya niyetliyim**					
**Seyahat sağlığı hizmetlerini sunabilecek becerilere sahibim.**					
**Seyahat sağlığı konusunda güncel bilgilere erişebilirim.**					
**Seyahat sağlığı hizmetleri sunarken diğer sağlık çalışanları ile iş birliği yapabilirim.**					

*Lütfen bu bölümde yer alan ifadelere katılım derecenizi 1 (Kesinlikle Katılmıyorum) - 5 (Kesinlikle Katılıyorum) aralığında derecelendiriniz.*Adsız Bölüm

*Anketi mobil cihazınızdan yanıtlıyorsanız soruları daha rahat görebilmek için lütfen ekranı yatay konumda tutunuz.

6. *

Her satırda yalnızca bir şıkkı işaretleyin.

	1 (Kesinlikle Katılmıyorum)	2	3	4	5 (Kesinlikl Katılıyorum
**Seyahat sağlığı hizmetlerini sunmak benim için kolaydır.**					
**Seyahat sağlığı hizmetlerini sunabilecek güvene sahibim.**					
**Seyahat sağlığı hizmetlerini sunmayı düşünüyorum.**					
**Seyahat sağlığı hizmetlerini sunabilecek eğitime sahibim.**					
**Seyahat sağlığı hizmetlerini sunmayı planlıyorum.**					
**Seyahat sağlığı hizmetlerini sunmama yardımcı olacak çalışan/çalışanlara sahibim.**					
**Seyahat sağlığı hizmetlerini sunabilecek zamana sahibim.**					

Lütfen aşağıda verilen davranışları gerçekleştirme sıklığınızı 1 (Hiçbir zaman) - 5 (Her zaman) aralığında derecelendiriniz.

*Anketi mobil cihazınızdan yanıtlıyorsanız soruları daha rahat görebilmek için lütfen ekranı yatay konumda tutunuz.

7. *Lütfen aşağıda verilen davranışları gerçekleştirme sıklığınızı 1 (Hiçbir zaman) - 5 (Her zaman) aralığında derecelendiriniz.*

Her satırda yalnızca bir şıkkı işaretleyin.

	1 (Hiçbir Zaman)	2	3	4	5 (Her zaman)
**Seyahat edecek hastalarım için sağlık risk değerlendirmesi yaparım.**					
**Seyahat edecek hastalarıma seyahatleri sırasında karşılaşabilecekleri sağlık sorunlarına ilişkin danışmanlık yaparım.**					
**Seyahat edecek hastalarıma bağışıklamaya ilişkin danışmanlık yaparım.**					
**Seyahat edecek hastalarıma seyahatleri sırasında ihtiyaç duyabilecekleri ilk yardım bilgilerine ilişkin danışmanlık yaparım.**					
**Seyahat edecek hastalarıma seyahatleri sırasında ihtiyaç duyabilecekleri ilaçlar konusunda danışmanlık yaparım.**					

Lütfen aşağıda verilen davranışları gerçekleştirme sıklığınızı 1 (Hiçbir zaman) - 5 (Her zaman) aralığında derecelendiriniz.

8. *Lütfen aşağıda verilen davranışları gerçekleştirme sıklığınızı 1 (Hiçbir zaman) - 5 (Her zaman) aralığında derecelendiriniz.*

Her satırda yalnızca bir şıkkı işaretleyin.

	1 (Hiçbir Zaman)	2	3	4	5 (Her zaman)
**Seyahat edecek hastalarıma kullandıkları ilaç dışı ürünler konusunda danışmanlık yaparım.**	⬭	⬭	⬭	⬭	⬭
**Seyahat edecek hastalarıma seyahatleri sırasında ihtiyaç duyabilecekleri ilaç dışı ürünler konusunda danışmanlık yaparım.**	⬭	⬭	⬭	⬭	⬭
**Seyahat edecek hastalarıma seyahatleri sırasında ihtiyaç duyabilecekleri koruyucu malzemeler konusunda danışmanlık yaparım.**	⬭	⬭	⬭	⬭	⬭
**Seyahat edecek hastalarıma seyahatleri sırasında ihtiyaç duyabilecekleri tıbbi dokümanları konusunda danışmanlık yaparım.**	⬭	⬭	⬭	⬭	⬭
**Seyahat edecek hastalarıma seyahatleri sırasında ihtiyaç duyabilecekleri tıbbi bilgilere nasıl erişebilecekleri konusunda danışmanlık yaparım.**	⬭	⬭	⬭	⬭	⬭

Demografik özellikler ve genel sorular

9. **Cinsiyetiniz?** *

Yalnızca bir şıkkı işaretleyin.

- Kadın
- Erkek
- Belirtmek istemiyorum

10. **Eğitim dereceniz?** *

Yalnızca bir şıkkı işaretleyin.

- Lisans
- Yüksek Lisans
- Doktora

11. **Meslekte geçirdiğiniz yıl?** *

____

12. **Bir yıl içerisinde genel olarak kaç hastaya seyahat sağlığı konusunda danışmanlık sunmaktasınız?**

Yalnızca bir şıkkı işaretleyin.

- 10 veya daha az
- 11-20
- 21-30
- 31 veya daha fazla

13. **Daha önce eczacıların seyahat sağlığı hizmetlerindeki rol ve sorumluluklarını ele alan bir eğitime/seminere katıldınız mı?**

Yalnızca bir şıkkı işaretleyin.

- Evet
- Hayır

14. **Seyahat eden hastalara yönelik olarak aşağıda sunulan durumlardan hangisi/hangileri ile ilgili daha önce danışmanlık hizmeti sundunuz? (birden fazla şık işaretleyebilirsiniz)**

Uygun olanların tümünü işaretleyin.

- [ ] İshal/Kabızlık
- [ ] Güneş yanıkları
- [ ] Sıcak çarpması
- [ ] Yol tutması
- [ ] Soğuk algınlığı
- [ ] Ateş
- [ ] Enfeksiyon hastalıkları
- [ ] Bulaşıcı hastalıklar / Salgın hastalıklar
- [ ] Cinsel yolla bulaşan hastalıklar
- [ ] Aşılar
- [ ] Ağız sağlığı
- [ ] Gıda zehirlenmeleri
- [ ] Böcek sokmaları
- [ ] Gebelik/Laktasyon
- [ ] Pediatrik hastalıklar
- [ ] Kronik hastalıklar
- [ ] Diğer: ____________________

Adsız Bölüm

15. ***Eczacıların seyahat sağlığı hizmetlerindeki rollerine ilişkin eklemek istediğiniz görüş ve önerileriniz varsa lütfen belirtiniz.***

Çalışmamıza katılım sağladığınız için teşekkür ederiz.

Bu içerik Google tarafından oluşturulmamış veya onaylanmamıştır.

Google Formlar
