# Supplementary material for: Cross-sectional study on community pharmacists’ behaviours in providing travel health services in Türkiye: a structural equation modelling approach
Source: BMJ Open. 2026 May 15;16(5):e112047. doi: 10.1136/bmjopen-2025-112047 (PMC13182400; doi:10.1136/bmjopen-2025-112047)
Supplement: Supplementary file 3 [file bmjopen-2025-112047.reviewer_comments.pdf]

PEER REVIEW HISTORY

BMJ Open publishes all reviews undertaken for accepted manuscripts. Reviewers are asked to complete a checklist review form and are provided with free text boxes to elaborate on their assessment. These free text comments are reproduced below.

ARTICLE DETAILS

Title (Provisional)

A CROSS-SECTIONAL STUDY ON COMMUNITY PHARMACISTS' BEHAVIORS IN PROVIDING TRAVEL HEALTH SERVICES IN TÜRKIYE: A STRUCTURAL EQUATION MODELLING APPROACH

Authors

Arslan, Miray; Şehitoğlu, Ayşe Çiğdem; Özçelikay, Gülbin

VERSION 1 - REVIEW

**Reviewer**	**1**
**Name**	**Yang, Caijun**
**Affiliation**	**Xi'an Jiaotong University School of Medicine**
**Date**	**30-Oct-2025**
**COI**	**None**

---

1. The study constructs its model based on the Theory of Planned Behavior (TPB), but it fails to explain why the potential impact of subjective norms on "attitudes toward PTHS" was not tested. In classic TPB, subjective norms typically act directly on "intention"; however, some studies also explore their indirect effects on "attitudes." While the study only mentions that subjective norms influence intention, it provides no justification for excluding the "subjective norms → attitudes" path from the model. Additional explanation is needed regarding the theoretical or empirical considerations that led to the omission of this path.

2. The study states that the survey consisted of "10 online pages" yet required only "10 minutes to complete"—a discrepancy that raises concerns about operational feasibility.

3. The study only included "pharmacists who provided THS to at least one patient annually," which introduces significant limitations:

• Lack of sample variability: Among pharmacists already providing THS, both "Behavior (B)" and "intention" had a median of 4, resulting in minimal differentiation between the two variables. This limits the study's ability to fully validate the predictive power of the "intention → behavior" pathway.

• Loss of critical information: Excluding pharmacists who do not provide THS prevents analysis of why some pharmacists fail to offer THS (e.g., insufficient perceived behavioral control, negative subjective norms). This omission weakens the model's ability to reflect real-world group differences and reduces its generalizability.

This is the key issue in this paper!

4. Ambiguous Wording of "Support" in Subjective Norms Items. The three items measuring subjective norms use the term "support" (e.g., "the government/other health professionals/community supports me in PTHS") without defining its meaning, leading to significant ambiguity. "Support" could be interpreted differently by respondents:

• Material/resource support (e.g., government-provided THS training or supplies);

• Policy support (e.g., official recognition of pharmacists' role in delivering THS);

• Attitudinal support (e.g., verbal approval from peers or physicians).

This ambiguity means responses may not reflect a consistent understanding of "subjective norms," reducing the validity of the subjective norms construct and biasing the model's results.

Based on your bias results, actually no insight can be get from this research

---

**Reviewer**	**2**
**Name**	**Bapolisi, Wyvine**
**Affiliation**	**Swiss Tropical and Public Health Institute, Epidemiology**
**Date**	**25-Feb-2026**
**COI**	**None**

Review "Modelling Community Pharmacists' Behaviors in Providing Travel Health Services"

This is an interesting topic in a country with a high travel density. Understanding the behavior of Community Pharmacists in providing Travel Health Services can help enhance the quality and availability of Travel Health Services in the Community in Turkey.

However, the manuscript needs improvement. Here are some suggestions:

General comment

There is a need to improve the flow between the introduction, methods, results, discussion, and conclusion. Components of the theory of planned behavior (attitudes, subjective norms, perceived self-control, and the Travel Health Services behavior) need to be defined. The operational definition of each term needs to be provided. In addition, some sentences need clarification. The suggestions provided can help.

The **title** can be clearer and catchier: authors can add the design or type of study, for example, Community Pharmacists' Behaviors in Providing Travel Health Services in Turkey using a Structural Equation Modelling, or something similar.

Abstract: Authors should consider revisiting the first sentence for clarity. It can benefit from additional work after the other sections are restructured.

Background

The manuscript uses a rich and varied literature to present the background. However, we think that the problem could be better presented. We suggest that the authors shorten the section on HTS services worldwide. One or two sentences can present that.

They should focus on the known picture in Türkiye. What is the data? How far are community pharmacists interfering with those services? Are there any data on this? If not, state that no data are available to the best of your knowledge, and this topic is underestimated, despite the fact that community pharmacists are at the frontlines. At the end of the introduction, readers should understand how community pharmacists intervene, why/how they need to be involved or the missed opportunity when community pharmacists are not delivering travel health services (optimally). Some of these points are already there, but the background needs restructuring with an emphasis on the problem. Elaborating more on the misconceptions or the real problems resulting from involving community pharmacists or their main challenges can help define the problem and drive through the rest.

Clarity: Some sentences need to be reformulated or placed at a later stage

P.4 64-65, for example, the authors stated "community pharmacists must be trained on this subject… to avoid this misconception"

Sentences like "…must be trained" should come either in the discussion or in the conclusion. It sounds more like a recommendation.

Page 4, 74-75: while the importance of THS is stated in the literature…, there is still a gap in the detailed evaluation of community pharmacists' behaviors.

Authors need to emphasize more on why they focused only on community pharmacists, and excluded nurses and doctors. It can be a personal choice, but it is good to clearly indicate and "defend" the choice of this specific category in this specific context.

Methods

We commend the authors who present the methods extensively.

Here are some suggestions to improve:

- Add a specific sub-section "Study population": the first sentence in the Discussion can shift to this section. Authors can also describe how they identified participants or who was

eligible to participate. This can also be added to the sample size calculation, but having a separate sub-section can be straightforward. Did the study cover the whole country?

- Also add a section on “Study Design.”
- Data Collection: It is well stated how the survey link was shared until reaching the Pharmacists' Chambers in Turkey, but how did it reach community pharmacists? Links shared via mobile phones, via emails, or via any other means? Were pharmacists invited to answer the question at those Chambers? How did it reach the Community pharmacists? Those details are important to inform data collection strategies, for example, to reach those in remote areas and maybe, if any, those with limited literacy, but also for replicability.
- It was stated that 11 files were discarded because of missing data. What were the characteristics of those participants? Were there any specific possible reasons the authors could think of to explain those missing values? Were there more in one subcategory or region?
- Lines 95-96, **based** on the TPB and relevant literature, is this what the authors meant?
- Lines 100-101: This sentence needs to be clarified.
- Lines 105-106 need to clarify. Twelve PhD pharmacists piloted the questionnaire; online or in person? Are they the ones who responded? If yes, why PhD students only? It could have been better to include at least one per category to test it on a representative or more balanced population. A PhD student can use 10 minutes, while another may require more time.
- When using TPB, it is always a good practice to define terms in the context of your study. PTHS behavior: What does it stand for? Same for Community attitudes, subjective norms, and perceived self-control. It can be good to introduce first the items related to each category before presenting the Hypotheses. This will help define the hypothesis in a good manner, but also help the reader to follow without terms that might be empty. It can be good to state, for example Attitudes were evaluated by xxx items defined in table xxx or you can cite the items. There was a tool tested and adjusted. It is better to clarify what items fall under attitudes, subjective norms, and perceived self-control.

How did you operationally define the behaviour of THS? Attitudes? Subjective norms and self-perceived control. This should appear either in the introduction or at least in the methods section. The reader mustn’t look for it. Was it a categorical variable, or what type of variable was it? The answer to this question and the definition of each component, as explained above, will help you decide and better define the method. Did you use the SEM or GSEM?

- Furthermore, it is good to state the hypothesis, even though it should be clarified, the authors should, at a further stage refers to their hypothesis and how they evolved. But I would suggest dropping this if the aim of the study is well introduced, and all terms are clearly defined, the flow will improve.
- Line 138, it is impossible to determine the population size. Why? The authors need to provide the reasons. No official record of community pharmacists?

- Sample size calculation: it is good to provide the formula but you need to indicate the meaning of each symbol and the value authors attribute to each variable and the source of the value, for example what p (prevalence) did they use and why, same for q, d etc… After that they can easily state in a clear sentence, given those estimations the minimal sample size calculation was 96.
- Lines 169-170: 11 surveys were discarded; 11 questionnaires may be.
- Limitations: There is no need to say that authors were expected more answers than what they received unless the minimal sample size was not reached. This part can be deleted.
- There is a need to improve the flow between the introduction, methods, results, and discussion and conclusion. The suggestions provided can help.
- Since there is a separate section on Discussion, the title on line 168, page 8, can just be Results.
- Ethical considerations: When did the participants sign the informed consent? How did the authors handle the identification information?

Results

There is a good effort in presenting the results. Here are some suggestions for improvements

- When presenting frequency, always add the percentage it represents. At some place only frequencies are presented or both. It is better to be consistent and keep both to give an accurate and complete overview.
- What would be interesting in this study it can be to inform if the services regarding diarrhoea, sunburns, or motion sickness were provided intentionally within THS or just a general routine. This can be of value if you were able to determine it
- There is a better way of presenting results. It is very important to present the results of the CFA, EFA or SEM, but they are just analysis tools; what were you measuring? Results subsections could be Sociodemographic results, correlation between x and x, etc., for example. In other words, what was measured through all these, or why did you choose to perform this analysis (not only because it was previously done elsewhere), what does it translate to in practice?
- One or two sentences introducing or linking one section to another will improve the flow. For example, in this instance, a few lines on how the results of EFA were important for CFA and then CFA for (G)SEM? The reader should be able to link and understand why it is important to provide those results. One sentence can do that every time. Another sentence can shortly describe how EFA built CFA and CFA was incorporated or used to determine the SEM model.
- For example, vaccination and other practices came to the main finding, but it was barely present or analysed in the results
- Information about validity and reliability. Authors should also specify what subtype of reliability was used. Check also the difference between validation and validity.

Limitations

The authors present some limitations to the study. It is good, in parallel to the limitations, to also present the strengths of their study.

"… Further work, including qualitative designs, is needed to identify more deeply the issues impacting community pharmacists' motivation and desire to provide THS." This sentence also highlights the need to clearly state and operationally define different variables in the TPB used. Was the behavior being motivation or desire? Current practice? If this is clearly defined, it helps analyse, write and convey a clear message. Otherwise, attitudes, subjective norms and perceived self-control are empty words.

Here also intervenes the question I raised above on data collection. Was the questionnaire delivered straight to the community pharmacists? Or it was through a third person feeling for them, etc. This section is supposed to highlight methodological areas for improvement or any circumstances that happened and could influence the results or their interpretation. But also, some preventive measures were taken to avoid the methodological limitations from affecting the results.

Conclusion

They are very strong pieces of information that have not been developed in the other sections, such as vaccination. The conclusion should be based on the findings presented. What are the key findings or takeaway messages and their implications?

We believe the authors should improve the flow and clarify some sentences and better define variables and outcomes.

VERSION 1 - AUTHOR RESPONSE

Comments from Reviewer #1

We would like to thank you for your comments on our manuscript. The Reviewer 1 recommended rejection of the article, but we oppose this recommendation.

Comment 1: *Reviewer 1 asked why the study didn't address the relationship between attitudes and subjective norms.*

Response: In fact, he answered his own question. As Reviewer 1 stated, this relationship wasn't addressed in the original Theory of Planned Behavior model. The theoretical basis of our study is outlined in the study and is based on this model. Therefore, unlike numerous studies that have employed this theory in the literature, this pathway doesn't need to be explained.

Comment 2: *The Reviewer one stated that "The study states that the survey consisted of "10 online pages" yet required only "10 minutes to complete"—a discrepancy that raises concerns about operational feasibility."*

> **Response:** We disagree because, during the survey's pilot, it was determined that 10 minutes was sufficient time to answer the questions. Furthermore, there was no time limit for participants to complete the survey. Detsils are provided in the methods section.

Comment 3: *The Reviewer stated: "The study only included "pharmacists who provided THS to at least one patient annually," which introduces significant limitations:*

• Lack of sample variability: Among pharmacists already providing THS, both "Behavior (B)" and "intention" had a median of 4, resulting in minimal differentiation between the two variables. This limits the study's ability to fully validate the predictive power of the "intention → behavior" pathway.

• Loss of critical information: Excluding pharmacists who do not provide THS prevents analysis of why some pharmacists fail to offer THS (e.g., insufficient perceived behavioral control, negative subjective norms). This omission weakens the model's ability to reflect real-world group differences and reduces its generalizability.

This is the key issue in this paper!"

> **Response:** Again, we disagree. The primary purpose of this article is to identify the factors influencing the behavior of pharmacists who offer this service. How can the behavior of an individual who does not offer this service be measured? Our goal is not to distinguish between those who do and those who do not. Our goal is to provide theoretical guidance for those who do not offer this service, as well as for those who do. To clarify the study's aims, they are emphasized at the end of the Introduction section.

Comment 4: *The Reviewer 1 stated that "Ambiguous Wording of 'Support' in Subjective Norms Items. The three items measuring subjective norms use the term 'support' (e.g., "the government/other health professionals/community supports me in PTHS") without defining its*

meaning, leading to significant ambiguity. "Support" could be interpreted differently by respondents:

• *Material/resource support (e.g., government-provided THS training or supplies);*

• *Policy support (e.g., official recognition of pharmacists' role in delivering THS);*

• *Attitudinal support (e.g., verbal approval from peers or physicians).*

This ambiguity means responses may not reflect a consistent understanding of "subjective norms," reducing the validity of the subjective norms construct and biasing the model's results."

> **Response:** This survey was administered in Turkish to Turkish community pharmacists. In Turkey, there is no material or financial support for pharmacists to provide such services. Our regulations comprehensively define pharmacists' roles; according to them, pharmacists play a role in travel health. We only agree that we should give more detail about subjective norm coverage in the manuscript and add the following sentences to the methods section to clarify it:
>
> "Ajzen (1991) defined subjective norm (SN) as the subject's perception of social pressure from individuals who are significant to them in performing the behavior in question [33]. In Turkey, there is no material or financial support for pharmacists to provide THS; however, Turkish regulations define pharmacists' roles comprehensively, and, according to these, pharmacists are known to play a role in travel health. In this study, perceived social support is evaluated within the framework of subjective norms using 4 items focused on community, government, colleagues, and health professionals' support (e.g., "*The government supports me in PTHS"*). In this regard, the second hypothesis of the study is (H_2) Community pharmacists' subjective norms toward PTHS positively affect their intentions toward PTHS."

<u>Comments from Reviewer #2</u>

We would like to thank you for providing your constructive and detailed review comments on our manuscript. The recommendations and advice have significantly enhanced the quality of the manuscript.

Comment 1: *The title can be clearer and catchier: authors can add the design or type of study, for example, Community Pharmacists' Behaviors in Providing Travel Health Services in Turkey using a Structural Equation Modelling, or something similar.*

> **Response:** Thank you for this comment. The title was changes as "COMMUNITY PHARMACISTS' BEHAVIORS IN PROVIDING TRAVEL HEALTH SERVICES IN TÜRKİYE: A STRUCTURAL EQUATION MODELLING APPROACH".

Comment 2: *Abstract: Authors should consider revisiting the first sentence for clarity. It can benefit from additional work after the other sections are restructured.*

> **Response:** We thank the reviewer for pointing this out. We have restructured the abstract.

Comment 3: *The manuscript uses a rich and varied literature to present the background. However, we think that the problem could be better presented. We suggest that the authors shorten the section on HTS services worldwide. One or two sentences can present that. They should focus on the known picture in Türkiye. What is the data? How far are community pharmacists interfering with those services? Are there any data on this? If not, state that no data are available to the best of your knowledge, and this topic is underestimated, despite the fact that community pharmacists are at the frontlines. At the end of the introduction, readers should understand how community pharmacists intervene, why/how they need to be involved or the missed opportunity when community pharmacists are not delivering travel health services (optimally). Some of these points are already there, but the background needs restructuring with an emphasis on the problem. Elaborating more on the misconceptions or the real problems resulting from involving community pharmacists or their main challenges can help define the problem and drive through the rest.*

> **Response:** We appreciate the reviewer's feedback. Accordingly, the Introduction section was rewritten in light of the reviewer's comments.

Comment 4: *Some sentences need to be reformulated or placed at a later stage P.4 64-65, for example, the authors stated "community pharmacists must be trained on this subject… to avoid this misconception" Sentences like "…must be trained" should come either in the discussion or in the conclusion. It sounds more like a recommendation. Page 4, 74-75: while the importance of THS is stated in the literature…, there is still a gap in the detailed evaluation of community pharmacists' behaviors.*

> **Response:** Thank you for this comment. Mentioned sentences were moved to the discussion.

Comment 5: *We commend the authors who present the methods extensively. Here are some suggestions to improve:- Add a specific sub-section "Study population": the first sentence in the Discussion can shift to this section. Authors can also describe how they identified participants or who was eligible to participate. This can also be added to the sample size calculation, but having a separate sub-section can be straightforward. Did the study cover the whole country?*

Data Collection: It is well stated how the survey link was shared until reaching the Pharmacists' Chambers in Turkey, but how did it reach community pharmacists? Links shared via mobile phones, via emails, or via any other means? Were pharmacists invited to answer the question at those Chambers? How did it reach the Community pharmacists? Those details are important to inform data collection strategies, for example, to reach those in remote areas and maybe, if any, those with limited literacy, but also for replicability. ... Sample size calculation: it is good to provide the formula but you need to indicate the meaning of each symbol and the value authors attribute to each variable and the source of the value, for example what p (prevalence) did they use and why, same for q, d etc… After that they can easily state in a clear sentence, given those estimations the minimal sample size calculation was 96.

> **Response:** We appreciate the reviewer's feedback. The data collection subsection was renamed "Study population and data collection". Additionally, all details about the study population and sample size were provided in the "Study population and data collection" and "Sample size calculation" sections.

Comment 6: *It was stated that 11 files were discarded because of missing data. What were the characteristics of those participants? Were there any specific possible reasons the authors could think of to explain those missing values? Were there more in one subcategory or region?*

Response: Thank you for this comment. Details about the missing value were provided at the beginning of the Results. Added sentences are read as follows: "11 questionnaires were excluded from the analysis due to missing responses, as participants did not complete many questions."

Comment 7: *Lines 95-96, based on the TPB and relevant literature, is this what the authors meant? Lines 100-101: This sentence needs to be clarified. Lines 105-106 need to clarify. Twelve PhD pharmacists piloted the questionnaire; online or in person? Are they the ones who responded? If yes, why PhD students only? It could have been better to include at least one per category to test it on a representative or more balanced population. A PhD student can use 10 minutes, while another may require more time.*

Response: Thank you for this comment. Mentioned sentences were rewritten. And more details were added about the pilot study. Details about the missing value were provided at the beginning of the Results. Added sentences are read as follows: "11 questionnaires were excluded from the analysis due to missing responses, as participants did not complete many questions."

Comment 8: *There is a need to improve the flow between the introduction, methods, results, and discussion and conclusion. The suggestions provided can help.*

Response: We appreciate the reviewer's feedback. We tried to improve the flow between sections.

Comment 9: *Ethical considerations: When did the participants sign the informed consent? How did the authors handle the identification information?*

Response: Thank you for this comment. The incorrect wording used here has been corrected, and the process of obtaining consent has been explained.

Comment 10: *When presenting frequency, always add the percentage it represents. At some place only frequencies are presented or both. It is better to be consistent and keep both to give an accurate and complete overview.*

Response: Thank you for this comment. The percentages were added.

Comment 11: *There is a better way of presenting results…*

Response: Thank you for this comment. We partly agree with the reviewer. Accordingly, the Results section was reorganized in light of the reviewer's comments.

Comment 12: *The authors present some limitations to the study. It is good, in parallel to the limitations, to also present the strengths of their study...*

Response: Thank you for this comment. We reorginized limitation section. Also, we added the study's strengths after the abstract, per the editor's suggestion.

Comment 13: *They are very strong pieces of information that have not been developed in the other sections, such as vaccination. The conclusion should be based on the findings presented. What are the key findings or takeaway messages and their implications?*

Response: We appreciate the reviewer's feedback. We reorginized conclusion section.
